# An Account of the Operation of Tracheotomy for the Relief of Croup—of Lithotomy under Etherisation, and of Amputation of the Left Half of the Upper Jaw

**Published:** 1852-04

**Authors:** Henry H. Smith

**Affiliations:** Surgeon to the St. Joseph’s Hospital, Philadelphia


*An account of the operation of Tracheotomy for the relief of Croup—of Lithotomy under etherisation, and of Amputation of the Left Half of the Upper Jaw.* By HENRY H. SMITH, M. D., Surgeon to the St. Joseph's Hospital, Philadelphia.

The attention of the profession having been recently called, through the American Medical Association, to the importance of forming accurate surgical statistics, in order to obtain a correct estimate of the value of certain operations, the following cases are submitted as a contribution to the facts already published, on the subjects mentioned at the head of this article. As the object of the paper is to place these results in such a position as may render them available towards the formation of statistics hereafter, the account of each operation will be presented very briefly.

I. *Tracheotomy performed unsuccessfully for the relief of membanous croup.*—C——y, æt. 19 months, a large healthy boy, has had several slight attacks of false croup. His brothers, æt. 7 and 4 years, have also been subject to the same complaint, and his cousin, æt. 8, died recently of membranous croup. Nov. 23d, 1851, C—— was attacked with symptoms of croup resembling those of the other children, and of a former attack, and was therefore treated by the parents with lamp oil and other simple means. The next day, as he was no better, a physician was sent for, who deeming the case a mild one, directed the use of Coxe's hive syrup until relief was obtained. On the 24th and 25th the child appeared better, but on the 26th was again so much oppressed as to require medical aid, when the physician again directed the hive syrup, which vomited it freely. On the 29th, the child continuing to be much oppressed, the parents became dissatisfied, the physician was dismissed, and other advice sought.

In the afternoon of the same day, Nov. 29th, I saw it. The voice was now nearly inaudible, the cough sharp and barking, the respiration oppressed, and it had all the other well marked symptoms of croup. Suspecting from the history and symptoms, that the case would prove to be an insidious attack of membranous croup, I at once examined the fauces. The half arches and entire pharynx were of a dusky red color, the tonsils slightly swollen, with one or two spots of lymph upon their posterior surface, whilst a patch of the width of the little finger was adherent to the middle line of the pharynx, extending downwards. I immediately directed a solution of the nitrate of silver, grs. xl. to the ounce of water, and swabbed the entire surface of the pharynx and top of the glottis, with a piece of sponge wet with this solution. The application of the sponge brought away a considerable amount of whitened mucus, the escape of which was facilitated by the vomiting induced from the administration of a teaspoonful of powdered alum, every 15 minutes, as advised by Dr. Charles D. Meigs of this city.* Thinking that the exhaustion and debility forbade the abstraction of blood, either locally or generally, the little patient was put under the free use of calomel; rubefacient and stimulating frictions were made on the throat: the temperature and moisture of the air of the chamber carefully watched, and directions given to repeat the emetic with the addition of a warm bath if required, in the interval of my visit. Seven hours subsequently the child seemed easier. For a day or two these symptoms alternated, and the treatment was so changed as to add to the former remedies the free use of mild expectorants, until Monday, Dec. 1st, when at 12 o'clock M., the child suddenly became worse, it having during these days taken xxiii. grs. of calomel, without any signs of salivation. The respiration was now very difficult, and accompanied by great distress and anxiety, the skin cold and damp, with other symptoms of impending suffocation. I at once advised the operation of tracheotomy, which was readily assented to by the parents.

At 2 o'clock P. M., assisted by my friend Dr. Ellerslie Wallace and several office pupils, I operated according to the plan recommended by Dr. Pancoast,† excising a portion of the 3d, 4th and 5th rings of the trachea, and keeping the opening dilated by means of a ring, formed of pewter, the hooks of which were inserted upon the edges of the wound. After the spasm consequent on opening the trachea had passed off, the mucous membrane was carefully examined, and presented a perfectly natural appearance, being free from all signs of false membrane, in consequence of which the prognosis was deemed favorable. The incisions required during the operation were followed by a copious hemorrhage, and required the application of several ligatures in order to arrest it.

* See Medical Examiner, Vol. I, p. 414, 1838.

† American Journal of Medical Sciences, Vol. 17, N. S., p. 307, 1849.

At 4 P. M., the child was asleep and breathing easy; and through the attention of Messrs. Rommel, Wellford and others of my private class, was now carefully watched, the mucus being wiped out of the wound as it collected, and every possible attention paid to its condition.

Dec. 2d, 9, A. M. The little patient was apparently doing well, and had nursed repeatedly, but the tracheal mucous membrane looked red and dry; patches of dry mucus or lymph were also seen upon the edges of the wound, whilst a thick ropy expectoration occasionally escaped through it in the efforts to cough. The dryness was relieved by the use of a camel's hair pencil, wet with glycerin and water.

At 10, P. M., the child was sleeping quietly, with a good prospect of recovery; at 2, A. M., its respiration became impeded by a collection in the trachea, which, owing to its viscidity, was wiped out with difficulty by the pencil, but after this it again went to sleep.

At 4, A. M., it had another attack of dyspnœa from a similar cause, and under this commenced to sink, and died at 6, P. M., just forty two hours after the operation.

The publication of a few successful cases having lately induced some who have read them to anticipate much success from the operation of Tracheotomy, even when not resorted to until the last moment, the account of the fatal cases becomes especially necessary, in order to guard against a false conclusion. Although this child had been suffering from the disease for eight days, there was no false membrane about the trachea at the point where it was opened, and both Dr Wallace and myself noticed the natural appearance of the lining membrane of the canal, and anticipated from this a favorable result. Yet with every care death ensued within 48 hours after the operation.

Having recently had occasion to examine the results as shown in the sum total of such cases as have been reported, it is made evident that the success of this operation is not encouraging; as out of 245 cases 188 have died, being more than three-fourths of the whole number operated on.

2. *Lithotomy performed while the patient was under the influence of an anæsthetic.*—Joseph ———, from Delaware, æt. 8

years, was brought to me by his parents on Jan. 5th, laboring under the symptoms of stone in the bladder. A pebble about the size of a grain of coffee, was also shown, as having been passed when he was but 18 months old. After a short interval of rest, I sounded him, but his cries and struggles rendered a positive diagnosis impossible. A few days were therefore allowed to elapse, during which the free use of the warm hip bath, together with alkalies and anodynes internally, afforded much relief. The child was, however, very pale, delicate and irritable, and having been freely indulged by his mother in every whim, very intractable, screaming most lustily as soon as I entered his room.

A consultation being called, I determined to etherize him before introducing the sound a second time; but owing to his struggles at the approach of any one except his mother, was compelled to have him held on the bed whilst the sponge was retained to his mouth. In a few minutes, however, he became perfectly relaxed and tranquil, during which he was carefully sounded, and a stone distinctly felt in the lower part of the bladder. When the effect of the anæsthetic passed off, the little patient had no recollection whatever of the event, nor did he subsequently show any constitutional disturbance from the introduction of the sound. A few days more being deemed requisite in order to allow the extreme irritability of the bladder to pass off, the constitutional treatment was continued, but no intimation given him of the proposed operation.

On Wednesday, Jan. 14th, after being induced to smell the sponge in his room, (which he now did very willingly,) he was thoroughly etherized, and whilst in that condition, was carried up stairs to the amphitheatre of the University of Pennsylvania, where he was again sounded, and the stone being distinctly felt, was placed in the position for lithotomy. I then immediately performed the lateral operation of lithotomy, opening the bladder by means of Physick's gorget, and extracting a stone weighing upwards of five drachms, when the patient being untied, was carried to his bed, and the effect of the anæsthetic allowed to pass off; he subsequently declaring that he knew of nothing being done to him until placed in bed, when the flow of urine over the wound excited his sensibility. During his convalescence no bad symptoms were apparent, except a hemorrhage on the fourth day after the operation, consequent on his having accidentally pulled off a a ligature that had been placed upon one of the perineal arteries. In five weeks he returned to his native place, and has since, I am told, been entirely well.

The propriety of resorting to anæsthetics in operative surgery, is a question which fortunately for humanity is now doubted by few. As a general thing, I employ one part of chloroform to five parts of ether in nearly every instance, though in a case of lithotomy which I performed in the winter of 1851* upon an adult, and from whom I extracted a large and very rough stone weighing ℥ij. grs. xvii., the use of an anæsthetic was deemed unadvisable, in consequence of the intemperate habits of the patient rendering it imprudent to give him the quantity necessary to etherize him.

3. *Amputation of the left half of the upper jaw.*—E. F——d, æt. 48, had been laboring under a malignant tumor of the antrum for about six months, which was increasing so rapidly as to render it necessary to submit the question of the propriety of removing it to a consultation. After carefully reviewing the peculiarities of the case, it was decided that though there was reason to suppose the disease could not be eradicated from his system, yet the patient's life might be prolonged for many months by the operation, as had been the case in the operation reported by Dr. Horner.† The precise nature of the disease, as well as the other doubtful circumstances being fully stated to the patient, he decided "to entrust himself to Providence and his surgeon," and to submit to the operation. Accordingly on the 25th of February, 1852, he was brought before the medical class of the University of Pennsylvania, seated in a chair with his head well supported, and the anæsthetic administered, it being decided to save him the pain of the external incisions, but not to operate upon the mouth until consciousness had partially returned, lest the blood, by escaping into the throat, might induce suffocation. The first incisor tooth being extracted, I cut through the cheek by a slightly curved incision which extended from near the middle of the left zygomatic arch to a point in the upper lip about three lines in advance of the angle of the

* See Medical Examiner, vol. 7, No. 4, p. 233.

† Medical Examiner, vol. vi. p. 16, 1850.

mouth. The upper flap being then dissected up as far as the edge of the orbit and the anterior orifice of the bony nose, the masseter muscle was separated from its attachments to the upper jaw, and the patient found to be free from the effects of the ether. A fine saw was then passed around and behind the body of the malar bone, and its attachment to the superior maxillary divided.

The anterior edge of the palate plate of the palate bone being then separated from its articulation with the palate process of the superior maxillary by cutting it with a strong knife, and the middle palate suture being also divided from behind towards the alveolar process of the bone, the latter was sawed through from the nostril to the mouth. The nasal process of the superior maxillary was next sawed across on a level with the edge of the orbit, and the lower part of the bone cut off from the margin of the orbitar plate, after which an elevator was passed behind the superior maxilla, and its attachment easily overcome. A few touches of the knife through some fleshy fibres then enabled me to remove the bone nearly entire, the remaining portion of the orbitar plate being excised subsequently from below. A branch of the internal maxillary bled most freely during the last part of the operation, and so did some other vessels in the flaps, especially the facial, but the hemorrhage was perfectly controlled by the ligature without resorting to the actual cautery. The wound in the mouth being tightly packed with lint, that in the cheek was accurately adjusted by points of the twisted and interrupted sutures, and healed throughout its greatest extent by the first intention. The patient, six days subsequently, was able to sit up in his chair, presenting but little evidence of the loss of so considerable a portion of the bones of his face, and two weeks afterwards returned to his home.

